# Targeting nonalcoholic fatty liver disease via gut microbiome-centered therapies

**DOI:** 10.1080/19490976.2023.2226922

**Published:** 2023-06-26

**Authors:** Mijra Koning, Hilde Herrema, Max Nieuwdorp, Abraham S. Meijnikman

**Affiliations:** a Departments of Internal and Experimental Vascular Medicine, Amsterdam University Medical Centers, Location AMC, Amsterdam, The Netherlands; b Amsterdam Cardiovascular Sciences Diabetes, Amsterdam, The Netherlands; c Amsterdam Gastroenterology and Metabolism, Amsterdam, The Netherlands

**Keywords:** NAFLD, fecal microbiota transplantation, bacteriophages, probiotics, postbiotics, NASH, obesity

## Abstract

Humans possess abundant amounts of microorganisms, including bacteria, fungi, viruses, and archaea, in their gut. Patients with nonalcoholic fatty liver disease (NAFLD) exhibit alterations in their gut microbiome and an impaired gut barrier function. Preclinical studies emphasize the significance of the gut microbiome in the pathogenesis of NAFLD. In this overview, we explore how adjusting the gut microbiome could serve as an innovative therapeutic strategy for NAFLD. We provide a summary of current information on untargeted techniques such as probiotics and fecal microbiota transplantation, as well as targeted microbiome-focused therapies including engineered bacteria, prebiotics, postbiotics, and phages for the treatment of NAFLD.

## Introduction

The prevalence of nonalcoholic fatty liver disease (NAFLD) has reached epidemic proportions, underscoring the need to unravel its pathophysiology and the risks associated with the condition. The presence of steatosis in the liver in the absence of significant fibrosis has long been considered a relatively benign condition. However, steatosis can progress into nonalcoholic steatohepatitis (NASH), possibly leading to fibrosis, cirrhosis, and hepatocellular carcinoma.^1^ On top of that cardiovascular diseases are the leading cause of death among patients with NAFLD.^2^ It is now widely accepted that liver fibrosis as a result of liver injury secondary to NAFLD, is a major prognostic predictor for liver-related and overall morbidity and mortality.^2,3^ Our understanding of the factors that determine disease progression has evolved, but we are still not able to identify those patients who will progress to a more advanced stage in the disease and those who will not.^1^ One of these factors involved in the pathophysiology of NAFLD that gained significant interest in the field is the gut microbiome.

The gut microbiome is an extraordinarily complex ecosystem, containing organisms that span several kingdoms.^4^ Since Antony van Leeuwenhoek reported “small animalcules very swiftly moving” in microscopy samples of his stools and dental plaque, tremendous amount of research has been performed.^5^ The gut and liver have a close relationship, with most of the blood from the small and large intestine reaching the liver first through the portal vein. Microorganisms, including the large number residing in the gastrointestinal tract, have a profound impact on physiology and the ability to influence general health and disease states.^6,7^ With the introduction of affordable high-throughput sequencing, interest in and identification of the role of the gut microbiota in modulation of host metabolism has grown exponentially.^4^ It is now crystal clear that the gut microbiota influences numerous physiological processes including aging, digestion, absorption, metabolism, and immune system development and function.^4,8^ Searching for a future therapeutic target in the gut for NAFLD was therefore reasonable. The scientific interest for NAFLD increased exponentially in the past decade, with over 1500 publications in the last five years. Numerous studies of those 1500 have reported associations between the gut microbiome and NAFLD. Indeed, high-throughput sequencing combined with machine learning methods such as Mendelian randomization and mediation analyses, suggest causal relations between the gut microbiome and NAFLD, without performing intervention trials. In addition, accumulating clinical trials in humans have shown a beneficial effect on disease parameters, whereas others have not. In the recently updated guidelines of the American Association of the study of Liver Disease for the clinical assessment and management of NAFLD included the gut microbiome in the cellular and molecular pathophysiology of the disease.^9^ Nevertheless, targeting the gut microbiome for the treatment of NAFLD has not been included in the guideline. Exciting new microbiome-based approaches are being developed that have the potential to positively impact all aspects of liver disease.

Here, we give an overview of the alterations in gut microbial composition in individuals patients with NAFLD and summarize gut microbiome-centered approaches as future therapy for individuals with NAFLD.

## A gut microbiome signature for NAFLD

Several cross-sectional human studies have shown a connection between changes in gut microbiome composition and the clinical manifestations of NAFLD severity, including simple steatosis (NAFL), NASH, and advanced fibrosis related to NAFLD, in both pediatric and adult populations.^10–17^ The gut microbiome of those with NAFLD tends to differ from that of healthy individuals at the phylum level, with a rise in the number of *Proteobacteria* and *Firmicutes* and a reduction in the presence of *Bacteroidetes*. A recent comprehensive review and meta-analysis investigating the changes in the composition of the gut microbiome in patients with NAFLD found a distinct pattern consisting of increased levels of *Escherichia, Prevotella*, and *Streptococcus*, and decreased levels of *Coprococcus, Faecalibacterium*, and *Ruminococcus*.^18^ The meta-analysis also revealed that body mass index (BMI) might play a role in altering the levels of *Faecalibacterium* and *Prevotella* in patients with NAFLD compared to healthy individuals. This discovery emphasizes the importance to correct for BMI. BMI should be considered a confounder since clinical trials often compare healthy lean individuals to overweight NAFLD individuals. The same applies for diet,^19,20^ which of course correlates with BMI. Furthermore, the changes in the levels of *Streptococcus* and *Faecalibacterium* are considered to be markers of increased systemic inflammation and therefore a good indicator of NAFLD progression.^18^ While finding differences in the gut microbiome between patients with obesity and early stage of NAFLD is challenging, the most striking findings in regard to the connection between alterations in the microbiome and the clinical symptoms of NAFLD has been in patients with advanced fibrosis stage 3–4.^21^ This advanced stage of fibrosis has been linked to a reduction in overall microbial diversity, which is primarily due to an increase in gram-negative bacteria.

Loomba et al.^11^ conducted a study to determine if there was a specific gut microbial pattern linked to advanced fibrosis in NAFLD. The study involved 86 patients with biopsy-proven NAFLD, 72 of whom had minimal or no fibrosis (stages 0–2) and 14 had advanced fibrosis (stages 3–4). Through metagenomic sequencing, the researchers found that 37 different bacterial species, including *Escherichia coli* and *Bacteroides vulgatus*, were present in different quantities in patients with minimal versus advanced fibrosis. The researchers used this information, along with individual age, BMI, and microbial diversity, to create a prediction model with an impressive area under the receiver operating characteristic curve (AUROC) of 0.936 for detecting advanced fibrosis. In a later study, Caussy et al.^15^ found seven key bacterial species, including *Bacteroides caccae*, *Escherichia coli*, and *Clostridium sporogenes*, that were strongly linked to advanced fibrosis in NAFLD patients. Another study led by the same group identified 27 discriminatory bacterial species linked to NAFLD-associated cirrhosis and validated the findings in a separate group of first-degree relatives.^16^ Combining the stool metagenome profile with individual age and serum albumin levels achieved an AUROC of 0.91 for detecting cirrhosis in a multi-national cohort of 163 adults.^16^

These promising results suggest that a fecal sample has the potential to provide a noninvasive method for detecting advanced fibrosis in NAFLD, potentially avoiding the need for more invasive methods such as liver biopsy in individual with a progressive form of NAFLD. However, it is essential to validate the results across different populations, as differences in biomarkers and technical methodologies may affect the results. Nevertheless, a universal unique microbiome signature for NAFLD in various stages of NAFLD seems challenging because the gut microbiome is a vast collection of trillions of microorganisms, and each individual has an unique microbiome. In fact, the gut microbiome exceeds the human genome by a factor of nearly 1000 (22 million genes identified in the gut microbiome versus 23,000 genes in the human genome).^22,23^ The variance in the human gut microbiome is therefore extensive.^24^ Most of the variance of the human gut microbiome is still unaccounted.^25–27^ Part of the variance in the gut microbiome may be stochastic, yet several intrinsic and extrinsic factors such as host genetics, disease state, immune health, diet, socio-economic status, location, and medication are known to determine individual gut microbiomes.^4,28,29^ These factors should be considered when finding a personalized signature for NAFLD in various stages of the disease, which has not been established yet in any study.

## Beyond bacteria in the gastrointestinal tract

Most microbiome research focuses on bacteria in the gastrointestinal tract as they are abundant and linked to various diseases.^30^ Archaea are a rare but metabolically active component of the microbiome, and their function may go beyond methane production.^31,32^ Although, there are fewer fungi compared to bacteria, they can still have a significant impact on bacterial populations and human health through direct competition, commensalism, or metabolite production. Recently, it was shown that patients with advanced NAFLD have a different composition in their fecal mycobiome compared to patients with mild disease and increased circulating anti-*C. albicans* IgG levels were observed in these patients’ plasma.^33^ Other studies have shown that the major fungal phyla are Ascomycetes, Basidiomycetes, and Zygomycetes. *Candida* spp. potentially contribute to poor outcomes in pre-cirrhotic and cirrhotic liver disease.^34,35^ There is a growing awareness of more unknown components of the gut microbiota, including the virome. The vast majority (>99%) of viruses in the gut microbiome are prokaryotic viruses, bacteriophages, or phages from hereon. Phages are the most abundant biological entities in the environment and the number of phages in the human gut is estimated to be similar to bacterial numbers in the human host.^36,37^ Phage DNA can disrupt bacterial genes and because phage DNA can carry genes that alter bacterial host function, phages have an impact on mammalian metabolism. Lang et al.^38^ studied the virome in a NAFLD population and found that patients with advanced stage of NAFLD and severe fibroses had a decreased diversity of bacteriophages in comparison to patients with NAFLD and mild fibrosis. In this study population, the most dominant bacteriophages were the *Lactococcus* phages and more specific in patients with advanced form of NAFLD the *Escherichia, Enterobacteria and Lactobacillus phages*. Phages are an attractive target for therapy since they are the natural predators of microbiota, also they can target specific strains of bacteria.^39^ Limitations are the small therapeutic range and safety of phages.^39,40^ The past few years the gut phageome has also been studied in other diseases related to NALFD, such as metabolic syndrome (MetSyn) and type 2 diabetes (T2D).^40–42^ Still, a role for phages in human health and the gut microbiome has yet to be established. A few limiting factors in the research field are challenges in the level of identification, culturing and lack of clinical trials.^43,44^ In addition, it remains unclear whether changes in the virome are the result or the cause of disease.^39^ In the future, larger and extended metagenomics studies should be done to investigate the virome, its alterations, and the relationship with NAFLD.

Notably, most human gut microbiome studies are limited to analyzing the fecal microbiome. The gastrointestinal tract is very heterogeneous, and although limited studies are performed with upper gastrointestinal tract samples (i.e., small intestine), it is known that gut microbial diversity increases toward the colon.^45^ Even in the colon, the diversity varies depending on the studied segment.^45^ The fecal microbiome is an end-product and a result of a dynamic process along the gastrointestinal tract.^4^ Certainly, it provides insights into the general shifts within the gut microbiome, but species that are dominant throughout the gastrointestinal tract are not always detected in the feces.^46^ Shalom et al.^47^ developed a ingestible device that collected samples throughout the human intestine. They collected 240 samples from healthy donors and compared genetic variation, phages, bile acids and host-proteome within the intestine to those in the stool. They found significant differences between the intestine and stool among above mentioned components of the gut microbiome. This underscores the need to sample along the gastrointestinal tract if we want to unravel the role of the gut microbiome in NAFLD.

**Figure 1.**
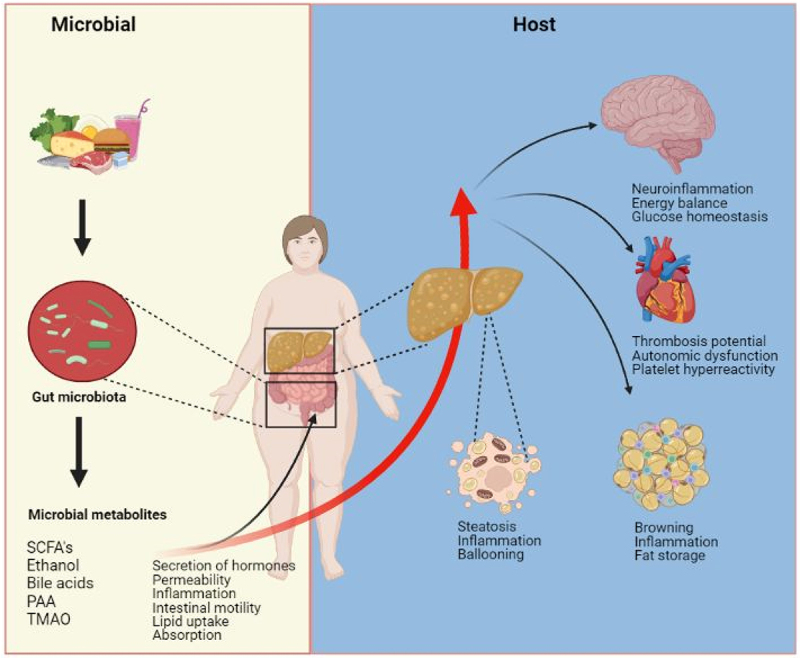
Metabolites produced by the microbiota produced via the diet-gut microbiota axis can have both local and peripheral effects in the host. The gut microbiota generates a variety of metabolites that can act either in the intestine or be absorbed into the host’s bloodstream and influence other organs. These metabolites can modulate the secretion of gastrointestinal hormones, which in turn can have peripheral effects. The liver is directly exposed to microbially produced metabolites through the portal vein and can metabolize some of them, resulting in the production of a distinct set of metabolites. The circulating metabolites can impact various organs in the body, causing changes in the host’s metabolism.

In addition to analyzing the presence, absence, or relative abundance of microbial species, functional analysis is an important aspect of microbial research. Changes in function are necessary to regulate the impact of the gut microbiota on health outcomes and drive therapeutic responses. Metabolic actions on distal tissues and organs by the gut microbiome are exerted amongst others via microbial metabolites.^48^ Human plasma serves as a liquid conveyor for molecules inside the body. The thousands of circulating small molecules, collectively called the plasma metabolome provides a unique insight into the interactions of genetics, lifestyle, environment, medication use and microbial activity.^49^ Notably, it was reported that approximately 60% of the variance in the plasma metabolome can be explained by the gut microbiome.^26^ The plasma metabolome can thus be used as a read-out of the functionality of the gut microbiome. Upon ingestion of nutrients, the gut microbiome determines which metabolites are formed and absorbed.^50^ Microbial metabolites modulate many key features of metabolic diseases such as insulin resistance^51^, platelet hyperreactivity^52^, thrombosis potential^53^, atherogenic lipid profile^54^ and ethanol production^55^ suggesting that the gut microbiome contributes to different metabolic perturbations that are associated with NAFLD (Figure 1).

## Targeting the gut microbiome

**Figure 2.**
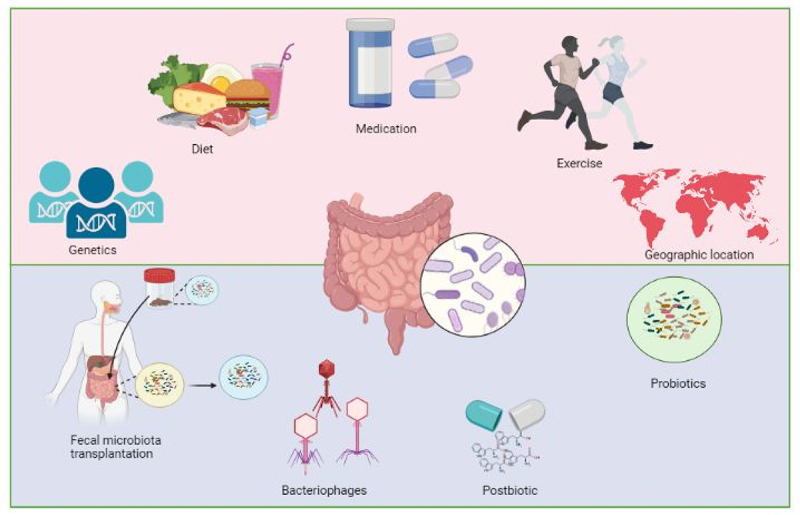
The composition of gut microbiota is influenced by various factors such as genetics of the host, dietary habits, medication, level of physical activity, and geographic location. Patients with nonalcoholic fatty liver disease have alterations in their gut microbial composition. To restore intestinal homeostasis, probiotics, fecal microbiota transplantation, bacteriophages and postbiotics can be used to restore the gut microbial composition.

Over the past few decades, there have been significant advancements in the field of gut microbial science. These include the discovery of the relationship between *Helicobacter pylori* and peptic ulceration^56^, the efficacy of fecal microbiota transplantation (FMT) to treat recurrent *Clostridioides difficile*^57^ infections, and the correlation between gut microbiome composition and responsiveness to checkpoint inhibitors in cancer.^58^ For several other diseases, the microbiome seems an attractive and feasible target as the effects are mostly restricted to the gut lumen and also have beneficial systemic effects. Here, we will discuss ways to target the gut microbiome that can be used as treatment for NAFLD (Figure 2).

## Fecal microbiota transplantation

The aim of FMT is to change a recipient’s microbiome for therapeutic purposes.^59^ A randomized controlled trial including 21 patients with NAFLD either receiving allogenic (donor) or autologous (own) FMT did not show any beneficial changes in regards of insulin resistance or hepatic proton density fat fraction on MRI. However, a significant improvement in intestinal permeability was observed following an allogenic FMT.^60^ We performed a proof of principle trial in patients with NAFLD whom either received an autologous or an allogenic FMT.^61^ A trend toward improvement of lobular inflammation and hepatocyte ballooning in the liver biopsy was observed after treatment with allogenic FMT. Considering that, from a clinical perspective, an FMT trial is successful if improvement or remission of a disease is achieved, this trial was negative due to being underpowered. However, from an ecological perspective the extent to which the donor’s microbiota can colonize the recipient microbiome is more important.^62^ A recent study, reanalyzing 316 FMTs derived from a wide range of indications, suggests, however, that clinical success is not dependent on colonization of donor strains, displacement of recipient species or the reinstatement of specific bacterial functions.^62^ This is line with the success of the autologous FMT, in both type 1 diabetes and inflammatory bowel disease.^63,64^ Predicting the outcome of FMT, from a clinical and ecological view remains difficult. Recent advances suggest that recipient factors are more important than donor factors, which is in contrast to the concept of super-donor’s, representing individuals with a highly diverse microbiome that were considered most effective FMT donors.^65^ Complementarity of donor and recipient microbiomes on community level and to specific strain population similarity are crucial for colonization and by proxy, clinical success. Matching donor-recipient microbiomes on community, species and strain levels could increase the success of colonization and hence clinical success and is therefore warranted.^62^ In addition, in these studies there was little focus on the mycobiome and virome, which complicates the understanding of interactions between the different ecosystems in the gut and thereby on success of the intervention.^33,36^ To further increase progress in facilitating the implementation of microbiome-based interventions, focus should be on unraveling these unknown factors. For a more in-depth examination of FMT in metabolic disease see other recent published work.^59^

## Probiotics and engineered microbes

Probiotics are defined as “live microorganisms that, when administered in adequate amounts, confer a health benefit on the host”.^66^ Probiotics come in a range of complexities but traditionally, probiotics consisted of single strains.^30^ While the positive impact of traditional probiotics like *Lactobacillus* and *Bifidobacterium* has been widely known for many years, it is only recently that researchers have started to understand the ways in which they work using rodent models. Supplementation of oral probiotics with *Lactobacillus fermentum* has been shown to reduce liver fat accumulation, oxidative stress, and levels of inflammation-causing markers (such as TNF-α and IL-18) in animal models with diet-induced NAFLD.^67,68^ Additionally, a mixture of *Bifidobacterium infantis, Lactobacillus acidopilus*, and *Bacillus cereus* has resulted in improved liver fat accumulation and inflammatory infiltration, as well as improved liver enzyme levels, serum LPS, and levels of inflammatory cytokines, in a high-fat, high-glucose diet animal model. The probiotic supplementation also increased the abundance of bacteria such as *E. coli* and *Enterococcus* while decreasing the presence of anaerobic bacteria such as *Lactobacillus, Bacteroides, and Bifidobacteria* .^69^ In humans, probiotics have shown limited potential to beneficially alter human metabolism. Two meta-analyses were conducted, encompassing a total of 28 clinical^70^ and 22 randomized-controlled trials,^71^ respectively, that included patients with NAFLD. The selection criteria for these trials involved testing the efficacy of probiotics in the treatment of NAFLD using radiological or histological evidence of fatty liver and excluding patients with a history of alcohol abuse. The results showed that probiotics reduced body mass index, liver enzyme levels, inflammation, and improved symptoms of diabetes and dyslipidemia.^70,71^ A major limitation however is the inconsistent use of various probiotics across different clinical trials, with some trials even combining probiotics with other substances and can even have severe adverse outcomes. The length of treatment in these trials can also vary widely, spanning from mere days to multiple years. Unfortunately, very few clinical trials have repeated the same probiotic regimen for comparison.

Advances in anaerobic culturing, in combination with improved sequencing technologies, enabled the production of strains for specific conditions and have resulted in a wider availability of strains and have been termed ‘next generation probiotics’.^72^ Strains derived from *Faecalibacterium prausnitzii* ^73^, *Bacteroides fragilis* ^74^, *Anaerobutyricum soehngenii* ,^75^ and *Akkermansia muciniphilia* ^76^ are abundantly studied, with the latter being most dominant in the field. Nevertheless, so far, human studies have failed to show a clinically relevant benefit of next generation probiotics use, especially in NAFLD. Considering that most of these strains are strictly anaerobic, the lack of clinical success is often explained by failure of engraftment because the viability is reduced in the small intestine.^77^ To overcome these challenges, the production of multiple bacterial strains into a single probiotic, whereby interactions can be directed to increase the success of engraftment or production of the desired metabolite are currently under development.^77,78^ These so-called multi-strain consortia or engineered microbes thereof increases the likelihood of achieving a specific clinical target.

An engineered microbe producing the anti-inflammatory cytokine interleukin (IL)-22, has beneficial effects on epithelial cells like hepatocytes and gut enterocytes.^79^ In a mouse model of ethanol-induced liver disease, engineered *Lactobacillus reuteri*-secreting mice IL-22 was administered, resulting in increased expression of antimicrobial molecules in intestinal epithelial cells, such as Reg3g, which prevented bacteria translocation from the intestinal lumen to the liver and reduced liver injury, steatosis, and inflammation caused by ethanol.^79^ This demonstrates the positive impact of IL-22-secreting *L. reuteri* on enhancing antimicrobial activity and improving host-bacteria interactions in preclinical models. Additionally, delivering the peptide hormone glucagon-like peptide 1 (GLP-1) to the gut via bacteria can increase insulin production in intestinal epithelial cells and boost systemic insulin levels.^80^ In summary, engineered bacteria hold promise as a tool for microbiota-based therapies. They have the potential to restore balance in both the gut and the body as a whole. However, the benefits seen in preclinical models have yet to be confirmed in clinical trials. Further research is needed to determine the best approach for treatment, including the appropriate pretreatment, mode of delivery and dosage, frequency, and duration of therapy. It is also important to note the microbiota can vary greatly among patients with chronic liver disease, a personalized approach to treatment is essential. Screening patients before starting therapy can help guide the use of engineered bacteria

## Prebiotics

Prebiotics are non-digestible food ingredients and influence the gut microbiome by stimulating the growth and activity of one or more bacteria in the colon.^81^ In 2017, the ISAPP made a consensus on the definition of prebiotics. They defined prebiotics as “substrates that are selectively utilized by host microorganisms conferring a health benefit.”^82^ Examples of prebiotics for humans are the oligosaccharides fructans and galactans. These oligosaccharides stimulate the growth of *Bifidobacteria*. Prebiotics also stimulate SCFA’s and prevent the colonization of pathogens.^83^ In rodents, prebiotics demonstrated some interesting results. The prebiotics Inulin and oligofructosaccharide (OFS) reduced *de novo* lipogenesis and decreased liver triglyceride content in animals.^84–86^ In individuals with NAFLD, only a few randomized controlled trials have been performed. One small study with OFS showed a small but significant reduction of aspartate aminotransferase in seven patients with NAFLD, but it did not show a significant reduction of steatosis.^87^ On the other hand, a clinical trial with 14 patients with NASH (NAS > 5) received OFS or placebo and the group who received OFS had a significant reduction of liver steatosis.^88^ Comparable to other microbiome-targeted therapies for NAFLD, there are promising animal studies, but evidence from humans is still scarce. In order to establish the effects of prebiotics on NAFLD, the field needs more RCTs.

## Postbiotics

The metabolites produced by microorganisms play a crucial role in shaping the interactions between the gut microbiota and its host, as well as among different bacteria. Microbial metabolites can serve as potential diagnostic and therapeutic indicators for NAFLD. However, there is still debate over which metabolites are specifically produced by microbes, the host, or both. The International Scientific Association of Probiotics and Prebiotics has recently revised the definition “postbiotics” and not every microbial metabolite is now included. Postbiotics are bioactive molecules produced by bacteria and are now formally defined as a “preparation of inanimate micro-organisms and/or their components that confer a health benefit on the host”.^89^ The preparation, however, is not alive or viable, preventing the chance of colonization. Hence, the possible health benefits conferred by the postbiotics depend on the regular intake to maintain the presence of the functionally bioactive molecules. Some of the most interesting microbially generated metabolites include phenylacetic acid, bile acids, tri-methylamine oxide (TMAO), tryptophan derivatives, imidazole propionate, ethanol, and short-chain fatty acids (SCFAs) but also pasteurized *A. muciniphila*, which has a positive effect on health, but is not examined yet in a NAFLD population.^77^ For a more in-depth examination of the role of microbial metabolites in metabolic disease including NAFLD, see other published works.^90,91^ Phenylacetic acid (PAA) is a microbial metabolite that is formed via a metaorganismal pathway with phenylalanine as a nutrient precursor. Hoyles et al^13^ including women with severe obesity but without diabetes, that women with NAFLD had an unbalanced branched-chain and aromatic amino acid metabolism, resulting in increased levels of PAA. Causality of PAA in NAFLD development was obtained by performing studies in rodents and cell-lines showing that PAA can induce inflammation and fat accumulation in the liver. SCFAs are produced from fermentation of complex fibers by the majority of gut bacteria.^90^ SCFAs activate G-protein-coupled receptors, regulate the immune system, and reduce oxidative stress through the suppression of histone deacetylases.^91^ The production of SCFAs, specifically acetate, propionate, and butyrate, varies among individuals and is influenced by dietary fiber.^8^ There is ongoing research on the impact of these compounds on obesity and NAFLD in preclinical studies. For example, treatment with the butyrate prodrug Tributyrin in mice decreased insulin resistance and hepatic steatosis.^92^

Generally, SCFAs are considered beneficial for the human host, and some studies have chosen donor stool samples enriched in SCFA-producing bacteria for FMT. However, with advancing liver disease severity, SCFA levels decrease, and only propionate and butyrate are taken up by the liver.^93^ Moreover, stool and serum levels of SCFAs may not accurately reflect its actual availability.^94^ Nevertheless, studies have shown changes in SCFAs in patients with metabolic disease and liver disease before and after FMT but not yet in the setting of NAFLD.^95–98^ Also, the use of SCFA enemas and intravenous butyrate has been investigated for obesity in adults and children with relative success.^99,100^

In line, the degradation of tryptophan, a complex amino acid, can occur through both human and microbial pathways and affect various organs.^90,101^ The microbiota can directly convert tryptophan into indole and its derivatives and can enter host metabolic pathways leading to formation of kynurenine or serotonergic compounds.^90,101^ Oxindoles, kynurenine-related compounds, and other tryptophan metabolites have been linked to poor outcomes in cirrhosis.^102,103^ However, in general it is believed that indole-related metabolites help to fortify the intestinal barrier in cases of liver disease caused by alcohol consumption.^104,105^ For NAFLD specifically, it has been shown that lower circulating levels of tryptophan and increased activity of enzymes related to tryptophan metabolism, such as indoleamine 2,3-dioxygenase 1 and 2 (IDO1 and IDO2) and tryptophan-2,3-dioxygenase (TDO), along with their resulting metabolites, are associated with an increased risk of inflammation and fibrosis in the liver. In addition, metabolites derived from the indole pathway can decrease inflammation through the NF-kB pathway, and suppress cytokine production, including IL-22, and are involved in regulation of the innate immune system.^106^

Finally, microbially converted bile acids (critical to human health and the development of metabolic diseases) can serve as signaling molecules which can activate receptors in the gut, liver, and adipose tissue.^107^ The production of primary bile acids (cholic and chenodeoxycholic acids) from cholesterol in the liver is a complex process controlled by the nuclear receptor Farnesoid X receptor (FXR) and its downstream targets, FGF15/19 in the intestine and small heterodimer partner 1 (SHP1) in the liver.^108^ Once secreted into the intestine, bile acids can be altered by the gut microbiota.^109^ Primary bile acids are transformed into secondary bile acids (deoxycholic and lithocholic acid) through 7α-dehydroxylation, a process carried out primarily by bacteria from the Firmicutes family.^109^ The levels of bile acids in the blood and feces differ in various stages of NAFLD and in end-stage liver disease, the total bile acid pool is reduced and with lower formation of secondary bile acids.^110,111^ The relation between secondary bile acids and health is U-shaped, with both low and high levels being associated with inflammation and damage to the intestinal barrier.^30^ Approaches to address this issue have been studied for both the intestine, such as the use of apical sodium-dependent bile acid transporter (ASBT) inhibitors and bile acid sequestrants, and the liver, such as the use of ursodeoxycholic acid (UDCA). ASBT inhibitors are a class of drugs that target and inhibit the activity of the ASBT protein, which is involved in the reabsorption of bile acids from the intestine. Inhibiting the activity of ASBT leads to increased fecal excretion of bile acids, which can help reduce the amount of bile acids in the liver and potentially improve liver function in patients with NAFLD.^112^ Due to its immunomodulatory, antioxidant, and antiapoptotic properties, UDCA has been utilized as a treatment for various liver diseases and was regarded as a viable therapeutic option for the treatment of NAFLD.^112^ Although UDCA has shown promise as a potential treatment for NAFLD, its efficacy and safety in this context are still being investigated. There is growing evidence of the effectiveness of Nor-UDCA, a UDCA analogue, in treating NAFLD, although its direct impact on the microbiome needs further investigation. Another approach to modulating the impact of bile acids is through the use of FXR agonists, which are being tested for their efficacy in treating liver diseases, including NAFLD, due to their ability to regulate the gut-liver axis, partly through changes in bile acid and microbiome composition.^113–115^

In line with previous work,^116,117^ we recently indicated that endogenous ethanol could play a role in NAFLD in humans.^55^ It is widely recognized that patients with NAFLD and alcoholic fatty liver disease (ALD) have similar histological features, such as liver steatosis and an abundance of large Mallory bodies, indicating a shared underlying pathophysiology.^118–120^ Already over 50 years ago, it was shown that the liver has a large capacity to clear ethanol from the portal vein before it reaches the peripheral circulation.^121^ Ethanol is metabolized in the liver to acetaldehyde, which is done via the enzymes alcohol dehydrogenase (ADH), CYP2E1 and catalase.^122^ Acetaldehyde is known for causing DNA damage and suppressing DNA synthesis and repair mechanisms which is associated with the development of malignancies. Ethanol itself induces inflammation leading to the release of reactive oxygen species. Also, acetaldehyde and ethanol both influence DNA methylation causing changes in the expression of oncogenes and tumor-suppressor genes, which is hallmark of cellular senescence, a new but old player in NAFLD.^123,124^ Low circulating plasma levels of endogenously microbial produced ethanol have been described by several groups.^116,117,125–127^ We recently showed that patients with NAFLD produce considerable amounts of ethanol but that the first pass effect of the liver obscures the levels of endogenous ethanol production.^55^ Ethanol in bacteria can be produced by a process called the mixed acid fermentation pathway. Of interest, especially in individuals affected by or prone to develop NAFLD, the end products of the mixed acid fermentation are examples of postbiotics. The mixed acid fermentation pathway is the biological process in which, under anaerobic conditions, sugars are converted into a complex and variable mixture of acids including lactate, acetate, succinate, formate and ethanol.^128,129^ This metabolic pathway is common in bacteria including Gram-negative and Gram-positive bacteria.^128,129^ The formation of these gut microbial metabolites depends on the presence of certain key enzymes in the gut microbiota and the amount of oxidized nicotinamide adenine dinucleotide (NAD+). The first step is a glycolysis reaction where glucose is converted into pyruvate and NAD+ which is reduced to NADH. Pyruvate is then converted into acetyl-CoA and subsequently via the enzyme Alcohol Dehydrogenase (ADH) and oxidation of NADH to NAD+, ethanol is produced. The variety in end products, dependence on NAD+ and that the balance between end products is not “fixed”, suggests that the process can be altered and thus shifted toward different end products when the environment or redox potential is changed. Thus, finding the right postbiotic to alter the redox balance and thereby reducing ethanol production warrants further research.

## Bacteriophages

Phages, which are viruses that infect bacteria, are typically categorized based on their structure and sequence.^30,36^ They display a wide range of diversity and can target a specific strain of bacteria or multiple genera. Phages can be classified as either virulent or temperate, depending on their lifecycle.^36^ Virulent phages go through a lytic cycle, in which they bind to the bacterial cell wall, inject their DNA into the bacterium, replicate their genomic nucleic acids inside the bacteria, and use bacterial machinery to assemble progeny virions. Phages then lyse the bacterial cell wall, releasing the newly formed phages into the environment to continue the cycle.^36^ On the other hand, temperate phages can introduce DNA, possibly containing drug resistance and virulence factors, into the bacterial cells, which then integrate into the host chromosome as prophages.^36^ These temperate phages can switch to a lytic life cycle under certain conditions such as (chemical) stress and nutrients.^36^ The phageome in feces have been shown to change in patients with NAFLD. Patients with cirrhosis of mixed cause had a similar phage diversity to healthy individuals.^130^ In patients with a more progressive form of NAFLD had lower viral diversity than those with less advanced NAFLD.^38^ There was a higher presence Escherichia, Enterobacteria, and Lactobacillus phages were more abundant in those with advanced NAFLD.^38^ The impact of changes in the intestinal virome on the bacterial microbiota, and how this may affect the progression of NAFLD, is still not known. Phages however may have potential applications in treating NAFLD. A case report demonstrated the connection between ethanol-producing *Klebsiella pneumoniae* and NAFLD, with the presence of this bacterium found in 60% of Chinese patients with NAFLD.^117^ Administration of ethanol-producing *K. pneumoniae* via oral gavage caused steatohepatitis in mice.^117^ Similarly, transplanting feces containing ethanol-producing *K. pneumoniae* from a NAFLD individual into germ-free mice resulted in NAFLD. However, eliminating the ethanol-producing *K. pneumoniae* strain through phage therapy before transplantation prevented the development of NAFLD, suggesting that phage therapy could reduce liver disease.^117^

The potential of phages to target bacteria to deplete certain species within the ecosystem has shown beneficial effects in patients with ALD by reducing the circulation of the detrimental protein cytolysin, via targeting the cytolysin producing bacteria *Enterococcusus feacalis*.^131^ The virulence factor and toxin cytolysin, found in *E. faecalis* was linked to the severity of liver disease and mortality in patients with alcohol-associated hepatitis.^131^ To prove that cytolytic *E. faecalis* are crucial in the development of ethanol-induced steatohepatitis, humanized mice were given phages orally. Targeting cytolysin-positive *E. faecalis* with phages led to a reduction in ethanol-induced liver injury, steatosis, and inflammation, indicating that lytic bacteriophage treatment can selectively mitigate ethanol-induced liver disease caused by cytolysin-positive *E. faecalis* in humanized mice.^131^ These studies showcase the crucial role that pathobionts play in the development of fatty liver diseases. The use of phages to eliminate these bacteria has been shown to reduce liver disease in preclinical models. Most phages have a narrow host range, meaning they infect closely related strains within (related) species, limiting collateral damage to the microbiome of the recipient, via infecting other species.^36^ This narrow range however, might also be a reason why the ability of phages to modulate the gut microbiome is limited.^36^

## Sex differences in NAFLD and the microbiome

In medicine, differences between men and women are not uncommon. Especially in cardiovascular and metabolic diseases, differences between men and women are established.^132^ Considering differences in sex in patients with NAFLD, men have a higher risk of developing NAFLD than women. Interestingly, postmenopausal women have a higher prevalence of NAFLD then premenopausal women.^133^ This suggest that estrogens have protective role in NAFLD. It makes sense since women are at risk to gain more weight, develop insulin resistance and dyslipidemia when becoming postmenopausal.^134^ These are all risk factors for metabolic syndrome and thus NAFLD.

Once NAFLD is confirmed, women have a higher risk of disease progression than men.^135^ Another difference between men and women with NAFLD is the development of cardiovascular disease. Women with NAFLD develop more cardiovascular events than men of the same age.^136^ Since the microbiome is associated with the development of NAFLD, the question remains; are there sex differences in the gut microbiome in patients with NAFLD and could this be a target for therapy? Studies with mice showed correlations between gut microbiota composition and sex hormones such as estrogen and testosterones.^137,138^ There are not many studies performed in humans to distinguish microbiome diversity between males and females. A study in Seoul confirmed the relationship between high estrogen and testosterone levels and gut microbiome diversity, but could not confirm any differences between sex and the gut microbiome.^139^ Shi et al.^140^ examined gut microbiome profiles of patients with NAFLD and found significant differences in microbiome profiles between men and women. They found that the α-diversity of the gut microbiome was increased in women in comparison to men. Also, among patients with NAFLD, taxa prevalence was decreased in men and increased in women.^140^ Unfortunately, which factors actually contributed to these differences remained unclear.

## Conclusion and perspectives

Almost all papers related to microbiome research end with a conclusion stating that the field should move from correlation to causation, from observation to mechanism and from cross-sectional to longitudinal studies. Yet, it is true. Translating the discoveries from studies performed in rodent models to humans has and always will be one of the biggest challenges for the microbiome field. To overcome this, more thorough characterization of the gut microbiome, (plasma and fecal) metabolome, and host response is needed using advanced preclinical models, different stages of liver disease, and larger, longitudinal individual cohorts. Further research is also required to determine the factors and mechanisms that make an individual susceptible or resistant to specific interventions. In line with patients with type 2 diabetes, it is becoming increasingly apparent that the molecular and cellular processes driving NAFLD are highly heterogenous from one individual to the next.^141^ Combine this with the high complexity of the gut microbiome and large variance between individuals, it will be difficult to find a one-size-fits-all treatment strategy for NAFLD. For example, individuals who lack bacteria that produce butyrate and are insulin-resistant may benefit from supplementation with probiotics or the missing microorganisms or the metabolite itself. On the other hand, those with high levels of harmful microbially produced metabolites like ethanol may have a better response to supplementation with inhibitors specifically created to target the microbial enzymes that produce these metabolites.^50^ The priority should be to identify first and then validate the mechanisms that contribute to the disease’s pathophysiology in humans, so that treatments can be tailored to each individual. To achieve this, it is crucial to perform comprehensive phenotyping of individuals using omics data both before and after intervention, which can then be used to predict their response to specific treatments. Long-term studies are needed to determine which therapeutic approaches result in lasting changes and positive clinical outcomes since some studies showing that gut microbiome modifications are temporary, with a return to baseline within a few weeks to months. The development of functional assays to test how individual microbes respond to interventions may hold the key to using the gut microbiome as a predictor of clinical outcomes.

Although there is no registered treatment for NAFLD yet, the future therapeutic landscape is enriched with an impressive range of agents with mechanisms of action that target different factors of the pathogenesis of NAFLD.^1^ With the accumulating evidence that the gut microbiome is more than a mere bystander in the development and progression of NAFLD, gut microbiome-centered therapies might have a place in future guidelines. However, treatment of chronic liver disease, including NAFLD, is complex and requires a multi-disciplinary approach, and a permanent cure through a gut microbiome-centered therapy alone is highly unlikely due to the multi-factorial nature of the disease. Realistic expectations of what can be achieved through microbiome modulation need to be established. Modulating the gut microbiome may be more effective as an adjuvant to current NAFLD treatments, rather than as a sole therapy. Currently, there is not sufficient knowledge based on scientific evidence on the dose, frequency, and route of gut microbiome modulation, including the small bowel microbiota, for NAFLD and this is highly warranted.

In conclusion, the human gut microbiome may play a significant role in the development and progression of NAFLD, although our understanding of the relationship between the gut and liver is still limited. We believe that there is not one unique microbial signature of disease phenotype in NAFLD. Understanding the role of the microbiome in NAFLD has important clinical implications, including the potential to develop microbiome-based interventions that can effectively reduce disease severity and slow the progression toward cirrhosis and its complications. Microbiome centered therapies such as engineered bacteria, postbiotics, and phages have mainly been tested in preclinical models. The effectiveness and safety of microbiome-based treatments must be evaluated through rigorous pharmacological studies and larger randomized controlled trials in individuals with NAFLD.
